# A methanolic extract of *Zanthoxylum bungeanum* modulates secondary metabolism regulator genes in *Aspergillus flavus* and shuts down aflatoxin production

**DOI:** 10.1038/s41598-022-09913-3

**Published:** 2022-04-09

**Authors:** Asmaa Abbas, Colin W. Wright, Nagwa El-Sawi, Tapani Yli-Mattila, Anssi M. Malinen

**Affiliations:** 1 Department of Life Technologies, University of Turku, 20014 Turku, Finland; 2 School of Pharmacy and Medical Sciences, University of Bradford, West Yorkshire, BD7 1DP UK; 3 Department of Chemistry, Faculty of Science, Sohag University, Sohag, 82524 Egypt

**Keywords:** Fungi, Antifungal agents, Transcriptomics

## Abstract

Aflatoxin B1 (AFB1) is a food-borne toxin produced by *Aspergillus flavus* and a few similar fungi. Natural anti-aflatoxigenic compounds are used as alternatives to chemical fungicides to prevent AFB1 accumulation. We found that a methanolic extract of the food additive *Zanthoxylum bungeanum* shuts down AFB1 production in *A. flavus*. A methanol sub-fraction (M20) showed the highest total phenolic/flavonoid content and the most potent antioxidant activity. Mass spectrometry analyses identified four flavonoids in M20: quercetin, epicatechin, kaempferol-3-O-rhamnoside, and hyperoside. The anti-aflatoxigenic potency of M20 (IC_50_: 2–4 µg/mL) was significantly higher than its anti-proliferation potency (IC_50_: 1800–1900 µg/mL). RNA-seq data indicated that M20 triggers significant transcriptional changes in 18 of 56 secondary metabolite pathways in *A. flavus*, including repression of the AFB1 biosynthesis pathway. Expression of *aflR*, the specific activator of the AFB1 pathway, was not changed by M20 treatment, suggesting that repression of the pathway is mediated by global regulators. Consistent with this, the Velvet complex, a prominent regulator of secondary metabolism and fungal development, was downregulated. Decreased expression of the conidial development regulators *brlA* and Medusa, genes that orchestrate redox responses, and GPCR/oxylipin-based signal transduction further suggests a broad cellular response to M20. *Z. bungeanum* extracts may facilitate the development of safe AFB1 control strategies.

## Introduction

*Aspergillus flavus* is a widely distributed, opportunistic, filamentous fungus that infects crops such as wheat, maize, peanuts, rice, cottonseed, and dairy products during pre-harvest and under storage conditions^1^. Upon infection, *A. flavus* has the potential to synthesize many secondary metabolites, including aflatoxins, cyclopiazonic acid, aflatrem, and kojic acid^2,3^. Aflatoxin B1 (AFB1), the most toxic of these compounds, is categorized as a group I carcinogen by the International Agency for Research on Cancer^4^. AFB1 and additional G-type aflatoxins are also produced by *Aspergillus parasiticus*. Because aflatoxins (AFs) pose a global risk to human and animal health, there is an urgent need for the development of new effective strategies for controlling aflatoxin contamination in crops.

The AF biosynthesis gene cluster in *A. flavus* consists of an array of 29 genes, including the pathway-specific regulator genes, *aflR* and *aflS*^5^. The level of AF production is coupled to fungal growth and development by the action of several global regulators. These regulators respond to environmental stimuli and coordinate the expression and activity of the AF pathway as well as other secondary metabolite (SM) pathways^6^. Prominent global AF biosynthesis regulators include the Velvet complex, which mediates the cellular response to light; the fungal development regulators, BrlA and AbaA; the oxidative stress-related proteins, AtfAB, MsnA and SrrA; and several cell signaling-linked proteins, including G-protein coupled receptors (GPCRs) and enzymes involved in oxylipin biosynthesis^5,6^.

One prominent approach for reducing AF levels in agricultural commodities is to introduce non-aflatoxigenic *A. flavus* or *A. oryzae* strains into the field to promote biological competition with aflatoxigenic strains^7,8^. This practice typically decreases AF levels at harvest; however, the method may lose efficacy if crop drying is delayed because of unfavorable weather or lack of infrastructure^9^. Chemical AF decomposition procedures for cleaning contaminated commodities have also been investigated^10,11^. Another studied AF prevention strategy relies on the use of natural products isolated from (e.g.) medicinal plants. These plant extracts contain aromatic secondary metabolites, such as alkaloids, flavonoids, saponins, glycosides, and tannins^12^. Several lines of evidence have demonstrated that some plant extracts have antifungal or anti-aflatoxigenic activities because they are rich in antioxidants^13,14^.

*Zanthoxylum bungeanum* Maxim (Rutaceae) is a traditional Chinese herbal medicine belonging to the genus *Zanthoxylum*, which comprises 250 species with a worldwide distribution^15^. Many chemical compounds have been isolated and identified from different parts of *Z. bungeanum*. The main classes of *Z. bungeanum*-derived bioactive compounds include alkaloids, terpenoids, flavonoids, and fatty acids^16^. Flavonoids are used broadly because of their antioxidant activity^17^. Interestingly, organic extracts of *Z. bungeanum* possess antifungal and antioxidant activity. Terpenoids are significant components of plant essential oils—the major source of their special flavor—and have antifungal activity^18^. The essential oil extract of *Z. bungeanum* inhibits the growth of bacteria and fungi, including *A. flavus*^19^.

The aim of this study was to determine whether methanolic extract fractions derived from *Z. bungeanum* pericarps exert antifungal and anti-aflatoxigenic activities. We identified extract fractions containing the highest amounts of phenols, flavonoids and antioxidants, and showed that they efficiently inhibited AF production by *A. flavus* and, at higher concentrations, also inhibited fungal growth. RNA-seq data indicated broad transcriptional changes in SM pathways, including the suppression of AF pathway regulators, after methanolic extract treatment. Expression of the AF pathway-specific regulator genes *aflR* and *aflS* was only modestly affected. However, several global SM regulator genes were significantly down- or upregulated, suggesting their participation in mediating the inhibition of AF production by *Z. bungeanum* extracts.

## Materials and methods

### Chemicals, aflatoxigenic isolate, and plant material

The sources of commercial chemicals are listed in Supplementary Material. The aflatoxigenic *A. flavus* isolate AF2653 used in this study was previously isolated from a corneal ulcer^20^. Dried *Z. bungeanum* pericarps were obtained commercially from China in November 2018 and stored at − 20 ℃ for ~ 6 months before extraction and fractionation. The plant material was processed in accordance with institutional, national, and international guidelines and legislation.

### Verification of *Z. bungeanum* material

To verify the identity of the purchased *Z. bungeanum* material, we employed previously characterized DNA barcodes ITS2 (internal transcribed spacer) and ETS (external transcribed spacer), which localize to ribosomal RNA genes^21^. Genomic DNA was extracted from 40 mg of *Z. bungeanum* pericarps using a GenElute Plant Genomic DNA Miniprep Kit (Merck, Darmstadt, Germany). ITS2 and ETS barcodes were amplified by PCR (primers listed in Table S1) and sequenced from both directions. Obtained ITS2 and ETS sequences were compared to reference sequences from different *Zanthoxylum* species^21^ by constructing neighbor-joining phylogenetic trees with MEGA 7.0^22^. As a complementary identification approach, we performed a standard nucleotide BLAST^23^ search of our ITS2 and ETS sequences against the GenBank nucleotide collection^24^.

### Plant extraction and fractionation

*Z. bungeanum* pericarps (280 g) were macerated in 500 mL of petroleum ether for 2 days at 22 °C, after which petroleum ether was removed by filtration through Whatman filter paper. Pericarps were then macerated again for 4 days in methanol, and the filtered extract was dried under reduced pressure using a BUCHI Rotavapor R-200 to yield 75 g of crude extract. Half of this crude methanol extract was chromatographed over 250 g silica gel 60 (0.040–0.063 mm, 1.09385.1000; Merck) in a chromatography column (45 × 300 mm). The silica column was then eluted successively with 12 increasingly more polar solvent mixtures consisting of varying ratios of hexane, dichloromethane, ethyl acetate and methanol (Table S2). The initial volume of each eluted fraction was 500 mL. The eluted fractions were dried under reduced pressure using a Rotavapor and stored at − 20 °C. Most of the research was done using fractions EA80 and M20, corresponding to fractions eluted from the silica column with a mixture of 80% (v/v) ethyl acetate and 20% dichloromethane (fraction 7 in Table S2) or 80% ethyl acetate and 20% methanol (fraction 9), respectively.

### Determination of total phenolic content, total flavonoid content, and antioxidant activity of plant extract fractions

Total phenolic content (TPC) of eight plant extract fractions was measured using the Folin–Ciocalteu test as previously described^25^. Briefly, 0.1 mL of each fraction (re-solubilized as 1 mg/mL in 50% methanol) was mixed with 0.75 mL of the Folin–Ciocalteu reagent (diluted tenfold with deionized water before use) and incubated for 5 min at 22 °C. Next, 0.75 mL of 7.5% (w/v) sodium carbonate was added and the mixture was incubated for an additional 60 min at 22 °C. The absorbance at 725 nm was then measured using a LAMBDA XLS spectrophotometer (PerkinElmer). A calibration curve was made using 0–0.6 mg/mL gallic acid in 50% methanol; TPC was calculated as gallic acid equivalents in milligrams per gram of *Z. bungeanum* fractionated extracts.

Total flavonoid content (TFC) was determined using the aluminum chloride method as described previously^26^. Quercetin standards (0–25 µg/mL) or test samples were prepared in methanol. Standard or sample dilution (1 mL) was mixed with 1 mL 2% AlCl_3_, agitated for 30 s, and left to stand for 15 min at 22 °C. The absorbance of the mixtures at 430 nm was then measured, and TFC was calculated as quercetin equivalents in milligrams per gram of extract.

Total antioxidant activity was determined using the ABTS assay as described previously^27^ and in Supplementary materials. This assay monitors the antioxidant-dependent reduction of ABTS·+ radical cation as a change in absorbance at 734 nm. *Z. bungeanum* fractions were prepared at 0.1–1 mg/mL concentrations in 50% ethanol; 0.9 mL of ABTS·+ working solution was added to 0.1 mL of sample and incubated for 6 min at 22 °C. Absorbance was then measured using Trolox as a standard. Experiments were performed in triplicate. The total antioxidant activity of each sample was expressed as percent inhibition (PI) as defined in Eq. (1):

1 $$PI= \frac{{\mathrm{A}734}_{\mathrm{ABTS}}\cdot+ -{ A734}_{sample}}{{\mathrm{A}734}_{\mathrm{ABTS}}\cdot+}\times 100\%,$$

where A734_ABTS·+_ is the initial absorbance of diluted ABTS·+ at 734 nm and A734_sample_ is the absorbance of the sample. The potency of each sample was expressed as the ability to scavenge 50% of ABTS radicals (IC_50_, in mg/mL).

### Determination of the chemical composition of plant extract fractions

The identities of the major compounds in the plant fractions EA80 and M20, which contained the highest amounts of flavonoids, phenols and antioxidants, were confirmed using HPLC-mass spectrometry (MS) fingerprint analysis. Each extract fraction was re-solubilized at 10 mg/mL in methanol and filtered through a 0.45-μm Millex HA membrane syringe filter (SLHAO33SS, Merck Millipore); 20 µL of the filtrate was injected onto a Zorbax SB-C18 HPLC column (250 mm × 4.6 mm, particle size 5 μm, Agilent Technologies, and the column was eluted at a flow rate of 0.8 mL/min at 25 °C using a Shimadzu FRC-10A HPLC system and a programmed gradient of solvent A and B mixtures as previously described^28^, with modifications. The chromatogram was monitored at a wavelength of 254 nm. Solvents A (0.5% (v/v) trifluoroacetic acid in deionized water) and B (0.5% trifluoroacetic acid in methanol) were prepared fresh daily and filtered as above.

After optimizing the separation between peaks, low-resolution mass spectroscopy was performed using an HPLC–ESI–MS system consisting of a SiELC Primesep 100 column (250 mm × 4.6 mm, 5 μm) connected to a binary HPLC pump system with a Waters 2695 Sample Manager. Samples were eluted with the same solvent gradient procedure described above, and compounds were detected using a Micromass Quattro Micro API mass spectrometer (positive or negative electrospray ionization [ESI–MS]). High-resolution mass spectroscopy, used for determination of molecular formulas, was performed by the Analytical Centre, University of Bradford, UK, on an Agilent LC–MS containing an 1100 series LC/MSD Trap SL. Calculated, accurate masses were obtained through https://www.chemcalc.org/main.

### Determination of antifungal activity of plant extract fractions

The effects of *Z. bungeanum* plant extracts on the growth rate of *A. flavus* fungi were determined using a previously described quantitative micro-spectrophotometric assay^29^, with modifications. Briefly, the percent growth inhibition exerted by plant fractions was measured by determining the optical turbidity (at 595 nm) of the fungal culture in yeast extract sucrose (YES) medium in 96-well plates at the beginning and after 48 h of incubation at 28 °C. Each assay well contained 20 μL of plant fraction (dissolved in 10% DMSO), 10 μL of *A. flavus* spore suspension (10^3^ spores/mL), and 70 μL of YES broth (4 g/L yeast extract and 20 g/L sucrose in purified water). Control cultures were supplemented with 20 μL 10% DMSO instead of plant fractions. The effect of each fraction concentration on fungal growth was determined using three independent culture wells (biological replicates). Relative fungal growth was calculated using Eq. (2):

2 $$Relative\, growth\, rate= \frac{\Delta T}{\Delta C},$$

where ΔC is the corrected absorbance of the control culture (DMSO instead of plant fractions) and ΔT is the corrected absorbance of the culture containing the tested plant extract. The corrected absorbance values were calculated by subtracting the initial culture absorbance values from the values measured after a 48-h incubation. Relative fungal growth rate as a function of plant extract concentration was fit to Eq. (3)

3 $$Relative \,growth\, rate \,or \,AFB1\, production =A1+ \frac{A2-A1}{1+{10}^{\left({Log}_{x}0-\left[extract\right]\right)\times p}},$$

using Origin software (OriginLab, USA), yielding the parameters Log_x_0, Hill slope (p) and top asymptote (A2). The bottom asymptote, A1, was fixed to 0 because high extract concentrations completely prevented cell growth. Finally, IC_50_ values (i.e., plant extract concentrations exerting a reduction in 50% growth rate) were calculated using Eq. (4):

4 $${IC}_{50}={10}^{{Log}_{x}0}.$$

### Determination of the effects of plant extract fractions on mycelium weight and aflatoxin B1 production

Culture tubes containing 1 mL YES medium and varying concentrations of M20 fraction were seeded with *A. flavus* (10^3^ spores/mL) and incubated for 48 or 120 h in the dark at 25 °C. Fungus disks (mycelium) were then scraped off the tubes, filtered from the culture medium, dried, and weighed. AFs were extracted from culture medium and quantified by HPLC using a protocol adapted from Ref.^20^ as described in Supplementary Materials. Experiments were performed using three independent cultures for each extract concentration (biological replicates). IC_50_ values for AFB1 production were obtained using Eqs. (3) and (4).

### RNA-seq analysis

The effects of plant extracts on *A. flavus* gene expression were determined using RNA-seq. Three test and control tubes containing 1 mL YES medium and either 0.25 mg/mL M20 fraction or corresponding concentration of DMSO (0.5%) were seeded with *A. flavus* spores (10^3^ spores/mL). The tubes were incubated for 4 days at 25 °C in the dark. Mycelia were then harvested, grounded to a fine powder in liquid nitrogen, and used for isolation of total RNA as described in the Supplemental materials. RNA was dissolved in TE buffer^30^. RNA samples used for RNA-seq had RNA integrity numbers (RIN) of 8.6–9.4. cDNA libraries were prepared and sequenced at Novogene (UK) using the Illumina platform and a paired-end 150-bp sequencing strategy.

### Reverse transcription quantitative PCR

Key gene transcription changes suggested by RNA-seq data were confirmed by selecting six genes in the AF pathway and four regulators for quantitative reverse transcription-polymerase chain reaction (RT-qPCR) analysis. Total RNA was prepared from six M20 extract-treated and six non-treated control cell cultures as described for RNA-seq analysis. cDNA was synthesized from 20 ng of total RNA using gene specific primers and quantified using a Luna Universal One-Step RT-qPCR Kit (E3005S; New England BioLabs) according to the supplier’s protocol. cDNA was quantified based on SYBR Green fluorescence, and the PCR program was run on an iQTM5 Real-time PCR detection system (Bio-Rad). Relative gene expression levels in M20-treated versus control samples were calculated according to the 2^−∆∆CT^ method^31^ using beta-tubulin (AFLA_068620) as the reference gene^32^. Primers for *hypC*, *aflQ*, *aflW*, *veA*, *ppoC*, *cat2* and beta-tubulin were adapted from Ref.^33^, and primers for *aflN*, *aflR*, *aflS*, and *brlA* were adapted from Ref.^34^ (Table S3).

### Bioinformatics and analysis of differentially expressed genes (DEGs)

Raw RNA-seq data (FASTQ format) were processed with fastp, which removes adapter and poly-N sequences, yielding clean data/reads^35^, and the Q20, Q30 and GC content of clean data were calculated. All downstream analyses were based on high-quality, clean data. Paired-end, clean reads were mapped to the *A. flavus* NRRL 3357 genome (EnsemblFungi genome assembly JCVI-afl1-v2.0; www.ebi.ac.uk/ena/browser/view/GCA_000006275.2) using HISAT2 software^36^. Quantification of gene expression was calculated according to Fragments Per Kilobase of transcript sequence per Millions base pairs sequenced (FPKM)^37^; FPKM ≥ 1 was set as the threshold for an expressed gene. Differentially expressed genes (DEGs) between control and test samples were analyzed using the DESeq2 R package^38^. The combination of an FPKM fold-change ≥ 2 (or |log2 (fold change)| ≥ 1) and adjusted *p*-value < 0.05 was set as the threshold for DEGs in FunCat^39^ analysis performed using FungiFun^40^.

### Statistical analysis

Statistical analyses were performed using Origin (OriginLab) or SPSS Statistics 28 (IMB Corporation). Plant fraction content (TPC, TFC and antioxidants) and fungal growth and AFB1 production as a function of plant extract concentration data were analyzed using one-way analysis of variance (ANOVA) with post-hoc Tukey’s HSD. The null hypothesis—that observable means in any fraction or extract concentration are equal—was rejected where *p* < 0.01. Data normality was assumed.

RT-qPCR data were statistically tested in linearized form (i.e., as 2^−(Ct, treated − Ct, control)^ values). The validity of the data normality assumption was confirmed by Shapiro–Wilk test, and the *p*-values of transcription level changes were calculated using a two-sided *t*-test. The criterion for statistical significance was set at a *p*-value < 0.05.

## Results

### Identification of *Z. bungeanum* and its extracts containing the highest amounts of phenolic, flavonoid, and antioxidant compounds

We verified the identity of the purchased *Z. bungeanum* pericarps by sequencing ITS2 and ETS bar codes as previously described^21^. Neighbor-joining phylogenetic trees constructed from ITS2 (Fig. S1A) or ETS (Fig. S1B) sequences both indicated that our sequences are more closely related to *Z. bungeanum* than other *Zanthoxylum* species (i.e., our ITS2 and ETS sequences formed monophyletic groups with reference to *Z. bungeanum* sequences). BLAST searches further confirmed our plant material as *Z. bungeanum*, returning known *Z. bungeanum* ITS2/ETS sequences as the best hits (Fig. S1C).

**Figure 1:**
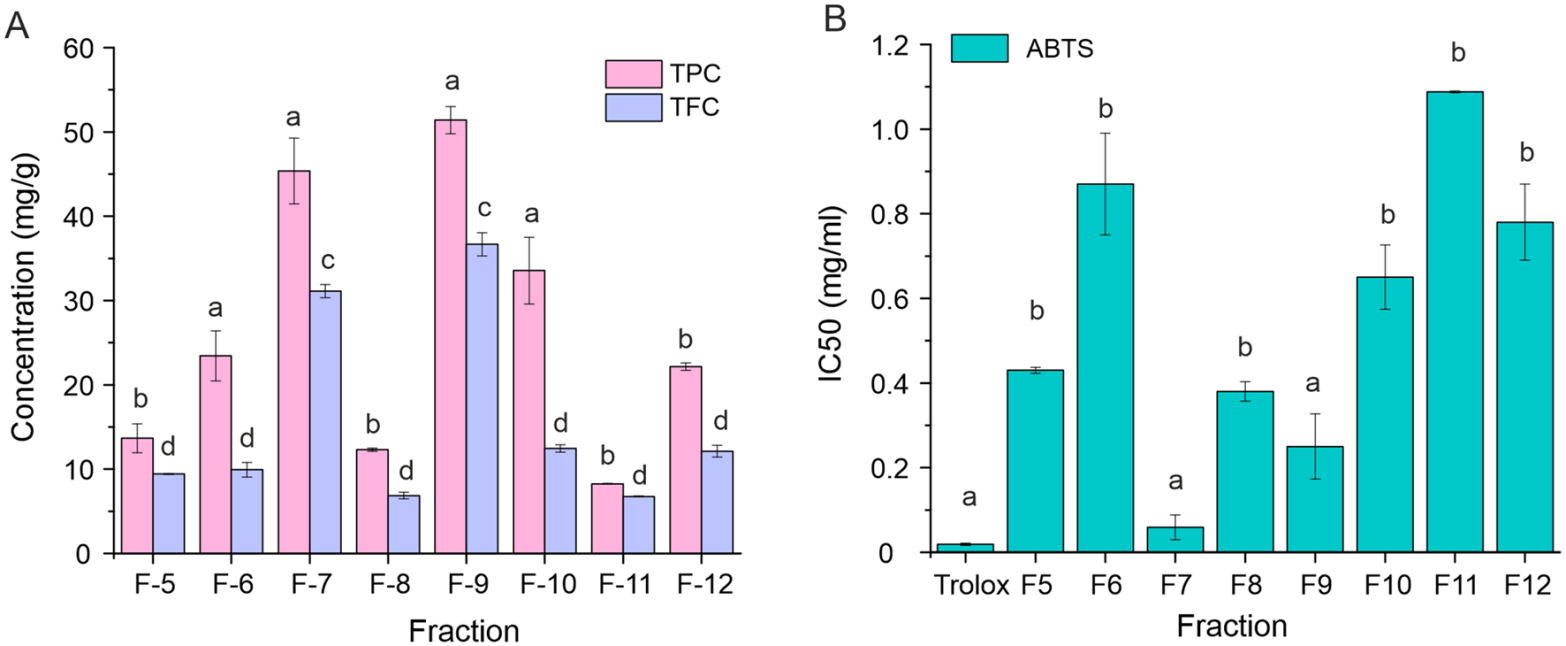
Phenols and flavonoids in plant extract. (**A**) Total phenolic content (TPC) and total flavonoid content (TFC) of isolated plant extracts F5–F12. (**B**) Antioxidant activities of Trolox standard and isolated plant extracts F5–F12 are shown as IC_50_ values. Data are expressed as means ± S.D (n = 3). Columns indicated with the same letter are not significantly different (*p* > 0.05) according to Tukey’s test.

Our aim was to determine whether *Z. bungeanum*-derived flavonoid compounds could inhibit AF production by *A. flavus*. If so—and if they could be used on a large scale to prevent AF contamination of food crops—they could potentially have significant health and economic benefits. To this end, we extracted and fractionated methanol soluble compounds from *Z. bungeanum* pericarps (Table S2). We then determined the total phenolic content (TPC; Fig. 1A), the total flavonoid content (TFC; Fig. 1A), and total antioxidant activity (Fig. 1B) of the fractions. Fractions 7 and 9, hereafter termed EA80 and M20, respectively, were found to have the highest TPC and TFC content (Fig. 1A). The IC_50_ values obtained from ABTS radical scavenging assays indicated that active antioxidant compounds were also most abundant in M20 and EA80 fractions (Fig. 1B). ANOVA with Tukey’s HSD test confirmed that EA80 and M20 groups together possessed the highest combined TPC, TFC and antioxidant activity.

### Identification of the main compounds in EA80 and M20 plant extracts

**Figure 2:**
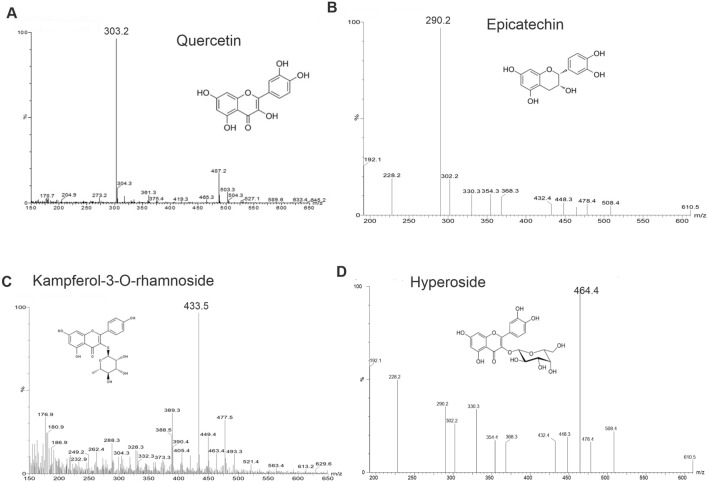
Identification of compounds in plant extracts. ESI–MS data for four main compounds isolated by HPLC from M20 and EA80 plant fractions. ESI–MS data were collected in positive ion mode. Molecular ions were observed either as a radical M+ or a protonated form [M + H]+. Based on mass-to-charge ratios (m/z), the compounds were preliminarily identified as (**A**) quercetin (m/z = 303.2, [M + H]+), (**B**) epicatechin (290.2, M+), (**C**) kaempferol-3-O-rhamnoside (433.5, [M + H]+), and (**D**) hyperoside (464.4, M+).

We identified the chemical constituents of EA80 and M20 fractions using HPLC–MS analysis. HPLC chromatograms revealed three abundant compounds in both EA80 and M20 fractions (Fig. S2). The molecular weights of these compounds, determined using ESI–MS, allowed preliminary identification of quercetin, epicatechin, and kaempferol-3-O-rhamnoside in both EA80 and M20 fractions (Fig. 2). In addition, hyperoside (hyperin, quercetin-3-galactoside) was found specifically in the M20 fraction. To confirm the identities of these compounds, we determined the molecular weights to an accuracy of up to four decimal places using high-resolution MS. These data confirmed the identity of all four compounds, yielding low mass-to-charge (m/z) deviations (Δppm 0.63–6.27) (Table S4).

### Antifungal and anti-aflatoxigenic activities of *Z. bungeanum* extract fractions

**Figure 3:**
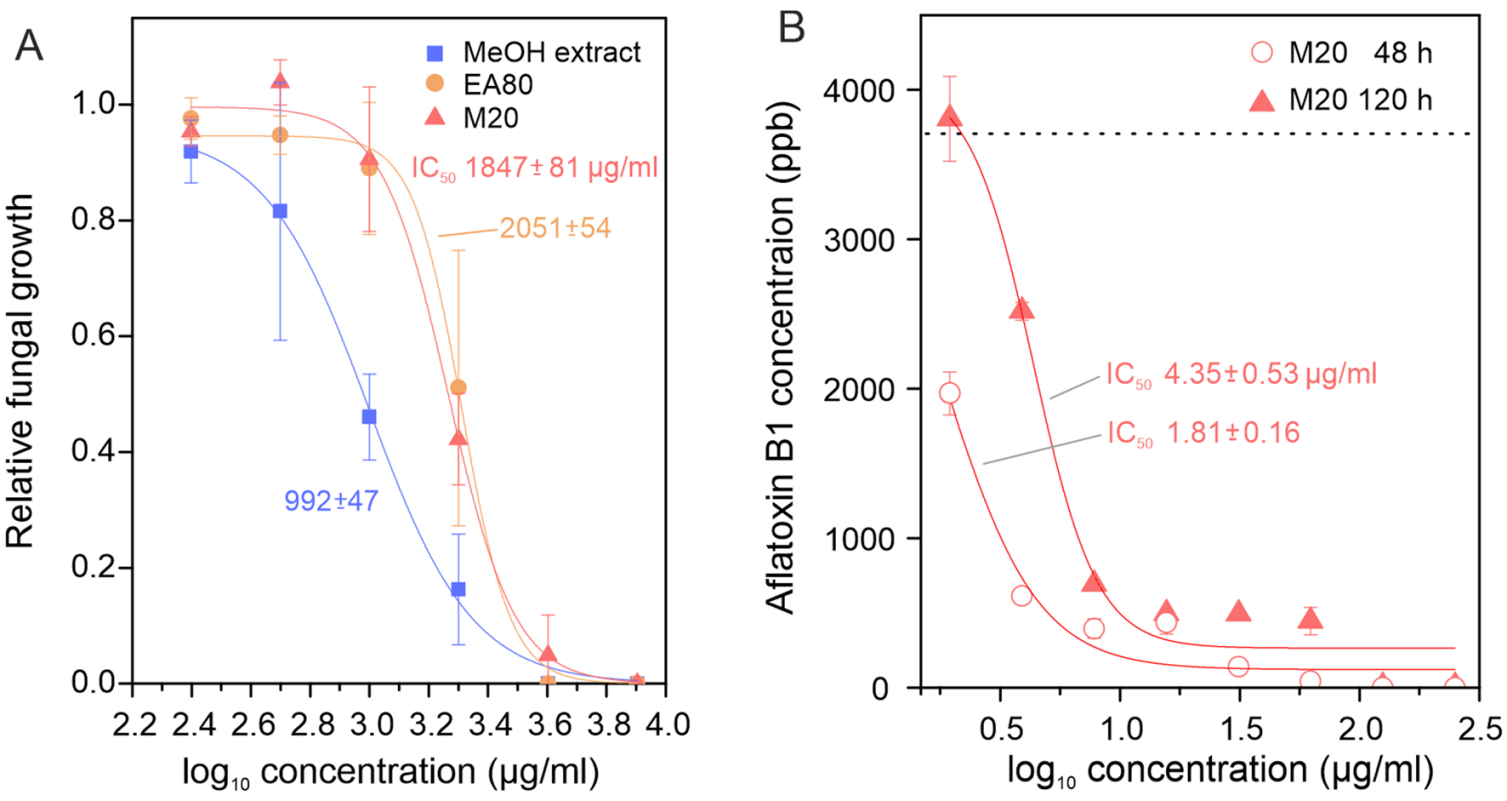
Antifungal and anti-aflatoxigenic activity of plant extracts. (**A**) Antifungal activities of crude methanol extract, and EA80 and M20 fractions of *Z. bungeanum*. Extract and fractions were used at 250, 500, 1000, 2000, 4000 and 8000 µg/mL concentrations. Relative *A. flavus* growth in liquid culture was estimated as optical density after 48 h culture. (**B**) Effects of the M20 fraction on AFB1 accumulation in *A. flavus* liquid culture medium after 48 or 120 h culture. M20 was used at 1.95, 3.9, 7.8, 15.7, 31.3, 62.5, 125 and 250 µg/mL concentrations. Dashed line indicates AFB1 production level in the absence of M20. Error bars in both panels are SDs for the averages of three biological replicates. Data were fit to Eq. (3), and IC_50_ ± SE values were calculated using Eq. (4). For all samples, ANOVA rejected the null hypothesis that all extract concentrations behaved the same (*p* < 0.001).

To investigate whether these extracts can inhibit cell proliferation, we treated liquid cultures of *A. flavus* AF2653 isolate^20^ with *Z. bungeanum* crude methanol extract, EA80 fraction, or M20 fraction. High concentrations (≥ 4000 µg/mL) of each extract fraction almost completely suppressed *A. flavus* cell growth (Fig. 3A). Based on their estimated IC_50_ values, the crude methanol extract appeared to be ~ twofold more potent than EA80 and M20 fractions (Fig. 3A).

To determine the effect of *Z. bungeanum*-derived compounds on AF production, we supplemented cultures of *A. flavus* in YES medium with the M20 fraction. Mycelium weight at the end of a 5-day culture period was only slightly affected (< 25% decrease), confirming that low M20 concentrations (≤ 250 µg/mL) have small effects on cell proliferation (Fig. S3). In contrast, M20 decreased the amount of AFB1 in the medium to non-detectable levels at concentrations as low as 125 µg/mL (Fig. 3B). The high potency of M20 as an inhibitor of AF biosynthesis is further highlighted by the fact that the IC_50_ value for AFB1 production (Fig. 3B) was 400–1000-fold lower compared with that for cell growth (Fig. 3A).

### Effect of treatment with the M20 plant extract fraction on the *A. flavus* gene transcription profile

We next employed RNA-seq to explore the molecular mechanisms by which extracts suppress AF production in *A. flavus*. Three independent cultures of control (no extract) and test (250 µg/mL M20) were assessed. At this concentration, M20 extract did not affect *A. flavus* proliferation but almost completely inhibited AF production (Fig. 3). RNA-seq produced 24.5–32.1 million clean paired-end reads (150 bp read length) per sample (Fig. S4A). Quality assurance statistics, such as low overall error rate (0.02–0.03%), high Q-score (94–95% bases in Q30 category) and invariable inferred GC content (52%), confirmed the high quality of sequencing data (Fig. S4A). Most reads (83–91%) were uniquely mapped to the reference genome of *A. flavus* NRRL3357 (Fig. S4B); gene exons were the most common mapping location (70–76%), followed by intergenic regions (22–28%) and introns (2%) (Fig. S4C). A Pearson’s correlation plot confirmed the expectation that differences in gene expression profiles were significantly greater between control and test groups (R^2^: 0.937–0.947) than among biological replicates within control (R^2^: 0.976–0.985) or test groups (R^2^: 0.985–0.987) (Fig. S4D).

**Figure 4:**
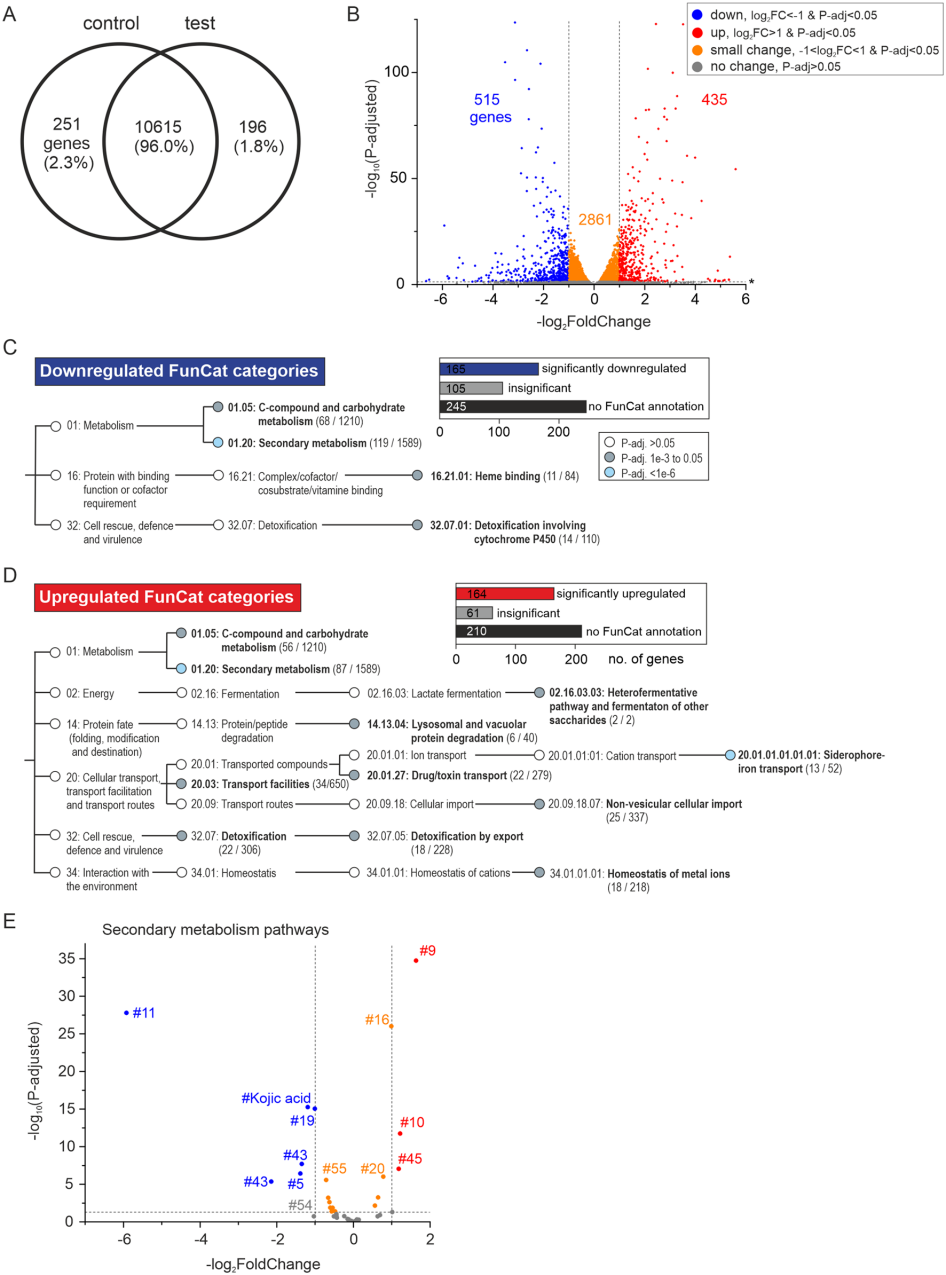
Effect of M20 plant extract on gene expression in *A. flavus*. (**A**) Venn diagram shows the number of genes expressed in both control and test groups (intersection) or uniquely in one of the two groups. (**B**) Volcano plot shows the classification of genes into downregulated, upregulated, small change or no change classes depending on their log_2_FoldChange and *p-*adj values. (**C**) FunCat categories that were significantly enriched among downregulated DEGs. Inset box shows the number of DEGs assigned as enriched (significant) or non-enriched (insignificant) in FunCat categories or lacking FunCat annotation. (**D**) FunCat categories that were significantly enriched among upregulated DEGs. Inset box is defined as in (**C**). (**E**) Effect of M20 treatment on SM pathways was evaluated by analyzing the expression of pathway “backbone enzyme” genes. Volcano plot shows SM pathways classified into downregulated (blue), upregulated (red), small change (orange) or no change (gray) classes depending on the log2FoldChange and *p*-adjusted values of backbone genes using limits detailed in (**B**). AF pathway is indicated by #54.

In total, 11,062 of a total of 13,485 genes (82%) in the *A. flavus* NRRL3357 reference genome displayed FPKM values ≥ 1 (average of three biological replicates) and were thus classified as expressed genes. Of genes classified as expressed, 96% (10,615) were expressed in both control and test groups, whereas 2.3% (251) and 1.8% (196) were expressed only in the control or test group, respectively (Fig. 4A). Using the combination of FPKM fold-change ≥ 2 and corrected *p*-value < 0.05 as selection criteria to identify differently expressed genes (DEGs) between control and test groups, we found a total of 950 DEGs (Fig. 4B). Among them, 515 genes were downregulated (Table S5) and 435 genes were upregulated (Table S6) in M20-treated samples.

We next analyzed the functions of identified DEGs by performing a FunCat pathways and categories^39^ enrichment analysis using FungiFun software^40^. About half of identified DEGs had a relevant annotation in the FunCat database, with 60–70% of the annotated DEGs showing enrichment for specific functional pathways/categories (Fig. 4C,D). Secondary metabolism as well as C-compound and carbohydrate metabolism were significantly enriched (*p*-adj < 0.05) among both downregulated (Fig. 4C) and upregulated (Fig. 4D) DEGs, suggesting that treatment of *A. flavus* with M20 extract modulates the expression of specific metabolite pathways within these broad FunCat categories. This enrichment analysis further indicated that M20 extract caused downregulation of cytochrome P450-dependent detoxification processes and heme-binding proteins (Fig. 4C). Upregulated DEGs, on the other hand, suggested stimulation of toxin export, lysosomal and vacuolar protein degradation as well as metal ion homeostasis—specifically, iron sequestration and import into the cell (Fig. 4D).

### Expression of SM pathways genes

FunCat pathway analyses indicated that treatment with M20 extract both downregulated and upregulated SM regulators (Fig. 4C,D). There are 55 predicted (by SMURF software^41^) SM gene clusters in *A. flavus*^42^, and at least one non-gene-cluster SM pathway that produces Kojic acid^43^. To decipher how individual SM pathways were affected, we analyzed the expression patterns of key “backbone” enzymes for each SM gene cluster as described in Ref.^44^ (Table S7). These backbone genes typically encode enzymes that catalyze an early committing reaction step in the SM synthesis mechanism^41^.

Analyses suggested that 20 SM clusters were transcriptionally silent in both control and treated samples (FPKM < 1 for the backbone gene; Table S7). Expression of pathway #7 and #26 was low in treated samples (FPKM 1.1–1.2) but was absent in the control group. The expression of eleven SM gene clusters was moderately changed (Fig. 4E, orange spheres; treated/control FPKM ratio (T/C) ≈ 0.5–2, *p*-adj < 0.05). The only verified SM product among these pathways was cyclopiazonic acid^45^, the product of pathway #55, which showed a 35–40% decrease in expression in treated samples. Three pathways were significantly overexpressed (T/C > 2, *p*-adj < 0.05) (Fig. 4E, red spheres). Experimentally verified products of #9 are aspergillicins, which are cyclic depsipeptides potentially involved in iron homeostasis^46^. The backbone gene of #10 is the scytalone dehydratase Arp1, suggesting that this pathway is involved in the biosynthesis of conidial pigments. The backbone gene of #45 is a putative nonribosomal peptide synthase, but the pathway product remains unknown. Treatment with M20 extract significantly downregulated (T/C < 0.5, *p*-adj < 0.05) four SM gene clusters and the kojic acid pathway (Fig. 4E, blue spheres). The product of cluster #11 is the antimicrobial mycotoxin, aspergillic acid. The products of other downregulated clusters are unknown. The backbone gene of #5 encodes polyketide synthetase PksP, which, based on its sequence homology to Alb1 protein in *Aspergillus fumigatus*, likely catalyzes the formation of a naphthopyrone compound^47^. The backbone genes of #19 and #43 encode putative dimethylallyl tryptophan synthases.

### Expression of AF biosynthesis pathway genes

**Figure 5:**
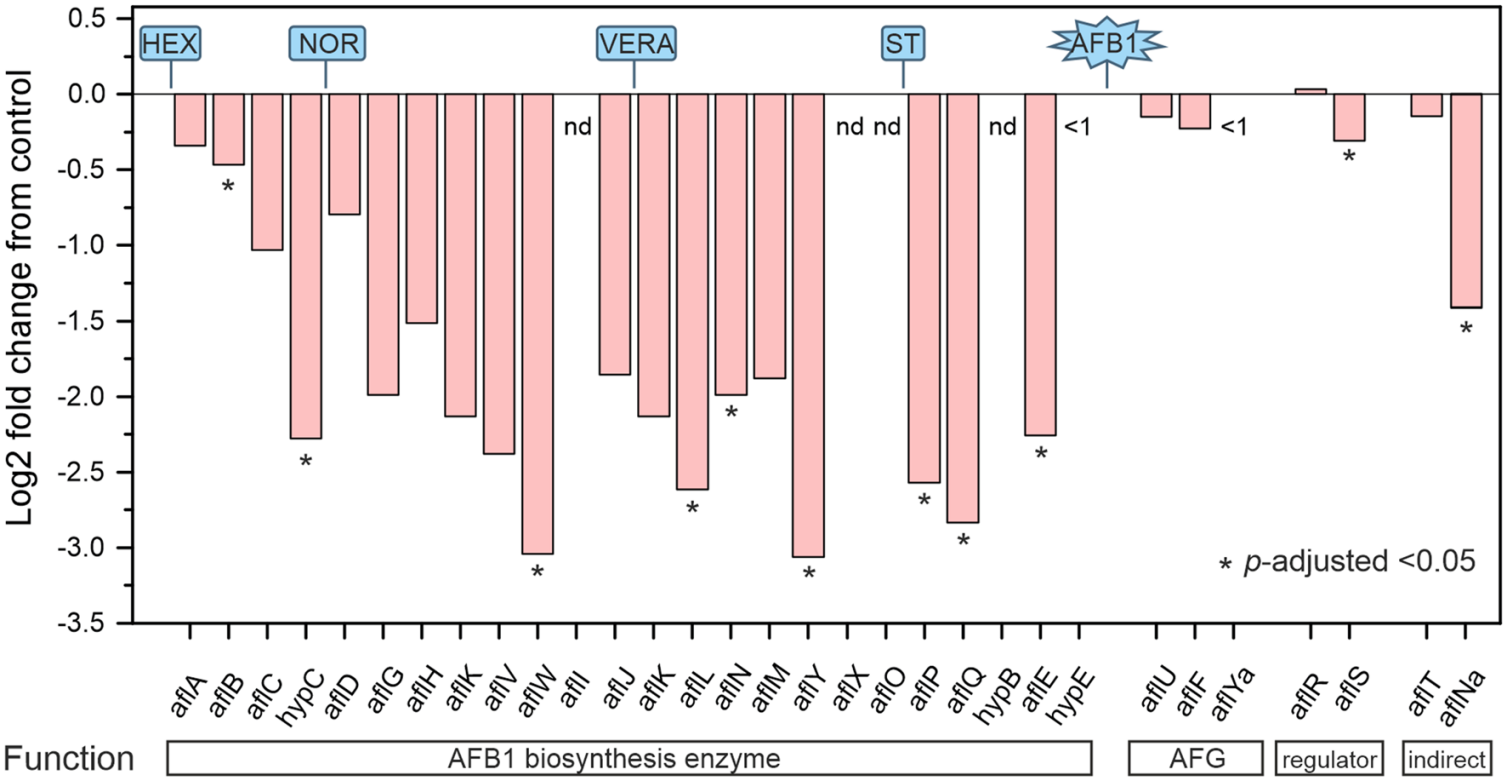
Effect of M20 extract treatment on AF cluster gene expression based on RNA-seq data. The names and reaction sequence locations of prominent chemical starter compounds (hexanoate, HEX) and intermediates, norsoloric acid (NOR), versicolorin A (VERA) and sterigmatocystin (ST), of the AFB1 synthesis pathway are shown. AFG indicates genes with a specific function in the synthesis of AF G-types. Genes absent in the reference genome annotation are indicated by nd. Genes with less than a threshold level of expression (FPKM < 1) are indicated by < 1.

We next assessed the effect of M20 plant extract on the expression of AF pathway genes by analyzing RNA-seq data (Table S8). AFs are the products of a complex polyketide pathway that consists of at least 27 enzymatic reactions^6^. AFB1 synthesis begins with hexanoate units (HEX in Fig. 5), which are derived from acetyl-CoA and malonyl-CoA, and proceeds via multiple intermediates, including the first stable intermediate norsoloric acid (NOR), through versicolorin A (VERA) and sterigmatocystin (ST) to mature AFB1. Consistent with the finding that M20 extract prevented AFB1 accumulation in *A. flavus* cultures, M20 downregulated all genes encoding enzymes directly involved in the AFB1 biosynthesis to at least some extent (Fig. 5, AFB1 biosynthesis enzymes). Nine statistically significant downregulated biosynthetic enzyme genes (out of a total of 19 in the reference genome) are spread across the AFB1 pathway. We re-analyzed four of these genes (*hypC, alfW, aflN* and *aflQ*) using RT-qPCR and found that all were significantly downregulated after M20 treatment, a finding in full accord with RNA-seq data (Fig. S5). RNA-seq data showed less impact on genes with specific functions in the synthesis of AF G-types (marked as AFG in Fig. 5). *aflT* and *aflNa* genes are located in the AF cluster but do not have direct recognized roles in AF biosynthesis^6^; of these two genes, a*flNa* was significantly downregulated. Interestingly, RNA-seq data showed no change in the expression of the cluster-specific essential transcription activator AflR, but revealed an approximately 20% decrease in expression of the cluster-specific non-essential co-activator AflS. RT-qPCR data confirmed the statistically insignificant change in *aflR* expression and showed a somewhat larger decrease (~ 60%) in *aflS* expression (Fig. S5).

### Expression of global AF regulator genes

To further explore the mechanism by which *Z. bungeanum* M20 extract shuts down AF production, we analyzed our RNA-seq data for changes in the expression of several previously characterized global AF regulator genes. In this section, we define a significant change in transcription as a treated/control FPKM ratio (T/C) ≤ 0.75 or ≥ 1.25 and *p*-adj ≤ 0.05.

Light level strongly affects SM production and fungal development. Velvet complex, consisting of the heterotrimer, VeA-LaeA-VelB, is the major light-responsive regulator in *Aspergillus* and has been suggested to control expression of the AF gene cluster and up to 27 other SM clusters in *A. flavus*^48^. We found statistically significant downregulation of *veA* and *laeA* (T/C ≈ 0.58 and 0.72, respectively), but not *velB* (Table S9). A similar degree of *veA* downregulation was confirmed by RT-qPCR (Fig. S5).

Carbon source affects AF production level in *A. flavus*. CreA, the master carbon-regulating transcription factor, together with its in-cell stabilizers, CreB and CreC, tune carbon utilization towards most favorable sources and directly control the AF pathway^6^. Notably, deletion of *creA* abolished AF synthesis in *A. flavus*. However, our RNA-seq data showed no significant change in *creABC* transcription levels (Table S9). Nitrogen source and regulators of nitrogen metabolism are also known to control AF production. Nitrogen utilization is mediated by nitrogen metabolite repression (NMR). *nmrA* negatively regulates several genes involved in NMR, and also appears to be involved in the regulation of development and AF biosynthesis in *A. flavus*^49^. Our RNA-seq data showed that expression of *nmrA* (T/C ≈ 0.72) and some *nmrA*-like genes, which encode NmrA-like protein domains, were significantly downregulated by treatment with M20 extract (Table S9). A low-pH medium promotes AF production^6^. However, expression of the principal pH-responsive transcription factor, *pacC*, did not significantly change in our samples (Table S9).

### Transcription of genes involved in fungal development and conidiation

Fungal development and asexual spore formation (conidiation) are tightly linked with SM production^6^. RNA-seq data showed that M20 treatment indeed repressed the expression of several genes associated with development and conidiation (Table S9). We observed a strong (~ fivefold) downregulation (T/C ≈ 0.2) of the gene *brlA* encoding the C2H2 zinc-finger conidiation transcription factor BrlA. This strong response was also confirmed by RT-qPCR (Fig. S5). BrlA is part of the BrlA → AbaA → WetA cascade, which is the primary regulator of asexual spore production in *Aspergillus*^50^; the expression of *abaA* and *wetA*, however, was not significantly affected. *brlA* promoter activity is governed by the concerted action of activators (FlbBCDE) and repressors (NsdD, VosA and VelB)^51^. M20 treatment caused a similar decrease (T/C ≈ 0.63) in transcript levels of the *flbD* activator and *vosA*/*nsdD* repressors, making it difficult to predict how these changes might integrate and contribute to the observed strong downregulation of *brlA* (Table S9). Among other conidium development-related genes, expression of the transcriptional regulator Medusa was decreased (T/C ≈ 0.70) whereas *fadA*, *fluG* and *hbx1* were not significantly affected (Table S9).

### Expression of genes involved in oxidative stress and antioxidant processes

Oxidative stress responses and SM production are considered to be tightly integrated^1^. Antioxidants inhibit AF biosynthesis, whereas compounds that cause oxidative stress have the opposite effect^13,14,44^. Several genes have been proposed to mediate the antioxidant-dependent inhibition of AF biosynthesis in *Aspergillus*^52^. A regulatory network consisting of the transcription factors Ap-1, AtfA, AtfB, MsnA and SrrA, as well as MAP kinase PbsB and adenylate cyclase AcyA, is involved in oxidative stress responses and SM production. RNA-seq data showed that, of these genes, the positive AF regulator *atfB*^53^ and co-regulator *atfA*^1^ were upregulated (T/C ≈ 1.50 and 1.42, respectively) in the M20-treated group, whereas another co-regulator, *srrA*^54^, was downregulated (T/C ≈ 0.75) (Table S9).

Catalases (CAT), superoxide dismutase (SOD) and glutathione peroxidase (GPX) enzymes convert reactive oxygen species (ROS) to less harmful products and thereby defend the cell against oxidative stress^55^. The activities of these enzymes and expression levels of their corresponding genes have been shown to increase in response to the AF production inhibitors piperine^56^ and cinnamaldehyde^55^. Our data showed a mixed anti-ROS enzyme response to M20 treatment. RNA-seq data showed that genes encoding mycelial catalase Cat1, the bifunctional catalase-peroxidase Cat2, and glutathione peroxidase Hyr1 were downregulated (T/C ≈ 0.27, 0.14 and 0.64, respectively), whereas the spore-specific catalase CatA and Cu/Zn superoxide dismutase SOD1 were upregulated (T/C ≈ 1.63 and 1.70, respectively) (Table S9). The strong downregulation of *cat2* was also supported by RT-qPCR results (Fig. S5).

A number of signal transduction pathways allow fungi to monitor and adapt to changes in environmental conditions. Among other components, these pathways involve GPCRs and hormone-like oxylipin molecules^57^. Oxylipin production depends on PpoA, PpoB and PpoC fatty acid oxygenases, which are under environmental control^58^. Deletion of the GPCR genes *gprK* and *gprA* increases AF production^57^, whereas deletion of *ppoAC* or *ppoABC* inactivates sterigmatocystin production (metabolically closely related to AF production) in *Aspergillus nidulans*^58^. Our RNA-seq data show no significant change in the expression of AF-related GPCRs after M20 treatment, whereas the expression of *ppoC* was strongly decreased (T/C ≈ 0.25) (Table S9). A similar degree of *ppoC* downregulation was confirmed by RT-qPCR (Fig. S5).

## Discussion

We found here that a methanolic extract of *Z. bungeanum* pericarps inhibits AF production by *A. flavus*. M20 and EA80 fractions, which contained the highest amounts of phenols, flavonoids and antioxidants, were studied in detail. We focused on these properties because of a previously reported correlation between the amount and identity of phenolic compounds and antioxidant capacity in plant-derived fractions^59^. M20 and EA80 shared three flavonoid compounds: quercetin, epicatechin, and kaempferol-3-O-rhamnoside; the M20 fraction contained the additional flavonoid, hyperoside. These flavonoids are known for their antioxidant activities and beneficial health effects^60^. The M20 fraction completely inhibited AFB1 production at a low concentration (≥ 0.125 mg/mL), whereas full *A. flavus* growth arrest required much higher concentrations (≥ 4 mg/mL; Fig. 3). Our data, and the finding that an aqueous extract of the medicinal plant *Micromeria graeca* inhibited AF production at a concentration of 0.6 mg/mL without affecting *A. flavus* growth^13^, indicate that AF production is generally more susceptible to chemical intervention compared with cell growth. Quercetin, the major compound in M20, has been reported to significantly inhibit AFB1 production, possibly by reducing ROS levels and thus oxidative stress^61^. We suggest that the anti-aflatoxigenic activity of the M20 fraction reflects the presence of flavonoids in general and quercetin in particular. The general anti-aflatoxigenic potential of *Zanthoxylum*-derived compounds is further highlighted by the recent finding that essential oils isolated from *Zanthoxylum armatum* inhibit both the growth and AFB1 production of *A. flavus* on stored platycladi semen^62^. Notably, none of the compounds identified in essential oils were found in M20 or EA80 fractions.

Using RNA-seq and RT-qPCR to obtain insight into the molecular mechanism of AFB1 inhibition, we found that genes in the AF gene cluster of *A. flavus* were generally downregulated by M20 treatment (Fig. 5). Significant gene downregulation was observed across the enzymatic reaction pathway, which converts hexanoate starter units via multiple intermediates to AFB1. AflR and AflS are the two AF cluster-specific transcriptional regulators required for efficient AF production. Indeed, *aflR* deletion leads to complete loss of AF synthesis^63^. Surprisingly, our data demonstrated that, although M20 treatment did not significantly affect *aflR* expression, it reduced the expression of *aflS* by ~ 20% (RNA-seq) to ~ 60% (RT-qPCR). These findings suggest that, instead of the pathway-specific regulators AflR or AlfS, some global transcriptional regulators are responsible for downregulation of the AF gene cluster. Downregulation of this cluster, however, is unlikely to involve widespread heterochromatin formation because FPKM counts of *aflR* (~ 70) and *aflS* (~ 200) indicate that these genes, which are located in the middle of a 70-kb long AF gene cluster^6^, were expressed at relatively high levels in all samples (Table S8). Consistent with the hypothesis that M20 treatment evokes a response by global secondary metabolism regulators, 18 of 56 SM pathways were differentially expressed, including downregulation of pathways that produce kojic acid, aspergillic acid or cyclopiazonic acid, and upregulation of the aspergillicins pathway (Fig. 4E). Cyclopiazonic acid is another mycotoxin produced by *A. flavus* that has been associated with different liver, kidney and gastrointestinal complications in veterinary medicine^64^.

**Figure 6:**
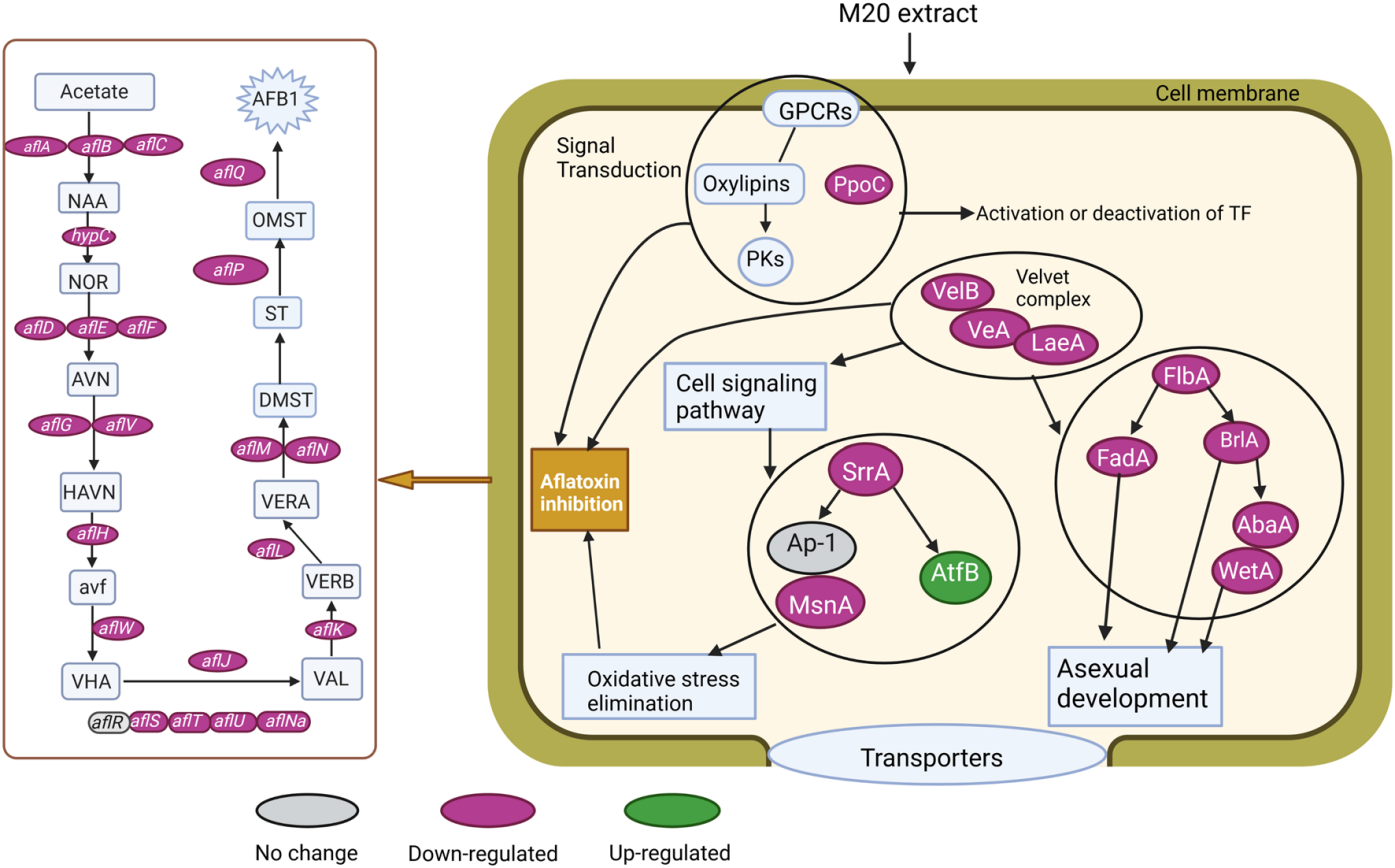
Schematic illustration of the anti-aflatoxigenic mechanism of M20 extract. In our working model, the extract causes downregulation of oxylipin-dependent cellular signaling, cellular development, the secondary metabolism regulator Velvet complex, and the oxidative stress pathway. Changes induced by M20 cause inactivation of the AF biosynthesis genes that mediate these processes, leading to a lack of the enzyme machinery required to produce AFs. *PKs* protein kinases, *TF* transcription factor.

VeA is a global transcriptional regulator that forms a trimeric Velvet complex with LaeA and VelB to modulate fungal development and conidiation, primary and secondary metabolism, and oxidative stress responses^65^. Our data indicated reduced expression of *veA* and *laeA* after M20 treatment and thus downregulation of the Velvet complex (Fig. 6, Table S9, Fig. S5). This may be the main reason for the absence of AF synthesis in M20-treated cultures. VeA also regulates expression of the early fungal development regulator *brlA* and the ratio of its α/β form transcripts^65^. Consistent with the observed downregulation of *veA* and Velvet complex, the expression of *brlA* was reduced by 80–90% (Table S9, Fig. S5). It was previously reported that benzenamine treatment downregulated *brlA* in *A. flavus*^66^, whereas treatment with eugenol^67^ or 5-Azacytidine^65^ upregulated the *brlA* gene. Nutritional factors, such as carbon and nitrogen source, and physiological conditions, such as pH, also affect AF biosynthesis in *A. flavus*^6^. Among the genes involved in these environmental responses, we found decreased expression of the nitrogen metabolism regulator *nmrA*, which has been proposed to act as a regulator of AF production^49^.

Antioxidant-related genes encoding superoxide dismutase (SOD) and catalases (CAT) are modulated by bZIP transcription factors as part of the cellular defense mechanism against ROS^56^. Here, we found that expression of *cat1* and *cat2* was highly reduced by M20 treatment, whereas expression of *sod1* and *catA* was increased (Table S9, Fig. S5). Consistent with this, exposure of *A. flavus* to cinnamaldehyde leads to higher SOD activity and reduced AF production^68^. Treatment with *M. graeca* extract was shown to repress AF production in association with downregulation of *cat1* and *cat2* genes^13^. GPCRs and their oxylipin ligands constitute signal transduction pathways involved in the regulation of AF production^6,55^. We found no response of the GPCR genes *gprA*, *gprP*, or *gprK* to M20 treatment, which contrasts with their stimulation by cinnamaldehyde^55^ or eugenol^67^ treatment. Oxylipin production is driven by Ppo fatty acid oxygenases. In this context, we found significant downregulation of *ppoC*, but unaltered expression of *ppoA* and *ppoB*.

In summary, we showed that simple organic *Z. bungeanum* extracts are enriched for flavonoids and inhibit AF production by *A. flavus*. Treatment with this extract induced significant transcriptional changes in *A. flavus* SM pathways, including repression of the AF pathway (Fig. 6). Repression of AF pathway was correlated with repression of global transcriptional regulators, including the Velvet complex, whereas expression of the main pathway-specific activator *aflR* was not affected. We thus suggest that the *Z. bungeanum* extract shuts down the AF pathway by depriving *A. flavus* of the essential transcription co-activation functions of global regulators. Collectively, our findings indicate that natural extracts from *Z. bungeanum* have potential to facilitate the development of safe and economical strategies for shutting down AF production in *Aspergillus* species.

## Supplementary Information

Supplementary Information 1.

Supplementary Information 2.

Supplementary Information 3.

Supplementary Information 4.

Supplementary Information 5.

Supplementary Information 6.

## Data Availability

RNA-seq data have been deposited in the NCBI Gene Expression Omnibus (GEO) database with the accession number GSE179477. Other data and propagable research materials are available upon reasonable request.
