# Supplementary material for: A methanolic extract of Zanthoxylum bungeanum modulates secondary metabolism regulator genes in Aspergillus flavus and shuts down aflatoxin production
Source: Sci Rep. 2022 Apr 9;12:5995. doi: 10.1038/s41598-022-09913-3 (PMC8994782; doi:10.1038/s41598-022-09913-3)
Supplement: Supplementary file 1 — Supplementary Information 1. [file 41598_2022_9913_MOESM1_ESM.pdf]

Supplementary Information

A methanolic extract of *Zanthoxylum bungeanum* modulates secondary metabolism regulator genes in *Aspergillus flavus* and shuts down aflatoxin production

Asmaa Abbas[1,2,3], Colin W. Wright[2], Nagwa El-Sawi[3], Tapani Yli-Mattila[1] and Anssi M. Malinen[1*]

[1]Department of Life Technologies, University of Turku, FI-20014 Turku, Finland

[2]School of Pharmacy and Medical Sciences, University of Bradford, West Yorkshire, BD7 1DP, UK

[3]Department of Chemistry, Faculty of Science, Sohag University, Sohag, 82524, Egypt

1. Detailed materials and methods

1.1. Source of chemicals

Petroleum ether, methanol, hexane, dichloromethane, ethyl acetate, acetonitrile, and sucrose were purchased from Fisher Scientific Company. Folin & Ciocalteu´s phenol reagent, gallic acid (98%), disodium carbonate, 2,2'-azino-bis (3-ethylbenzothiazoline-6-sulfonic acid) diammonium salt (98%), trolox (97%), potassium persulfate (K2S2O8), quercetin, aluminum chloride, trifluoroacetic acid, dimethyl sulfoxide (DMSO), and aflatoxin b1 (#A6636) were purchased from Sigma-Aldrich. Trizol and RNase/DNase free water were purchased from Invitrogen^{TM}. Isopropanol (RNase/DNase free) and Chloroform were purchased from Acros Organics. Yeast extract was from Neogen.

1.2. Determination of antioxidant activity of plant extract fractions

Total antioxidant activity was determined using the ABTS assay [27]. In this protocol, the ABTS•+ radical cation was generated by oxidizing 7 mM ABTS with 2.45 mM potassium persulfate. The radical is reduced back to ABTS by antioxidants in the sample with a concomitant decrease in the absorbance at 734 nm (where ABTS•+ absorbs strongly). The working solution of ABTS•+ was stored in the dark at room temperature for 12–16 h before use. The ABTS•+ concentration was adjusted to produce an initial absorbance value 0.7 at 734 nm. Five concentrations (0.1, 0.2, 0.4, 0.8 and 1 mg/mL) of different *Z. bungeanum* fractions were prepared in 1:1 ethanol:water mixture. 0.9 mL of ABTS•+ working solution was added to 0.1 mL of sample and incubated 6 min at 22°C before measuring absorbance at 734 nm. The total antioxidant activity of each sample was expressed as percentage inhibition (PI) as defined in equation (1) in the main text.

1.3. Determination of the effect of plant extract fractions on mycelium weight and aflatoxin B1 production

To determine the effect of plant extracts on the *A. flavus* cell mass accumulation and aflatoxin production, the test and control cultures in 1 mL of YES medium in micro-centrifuge tubes were seeded with spores (10^3 spores/mL) of aflatoxigenic *A. flavus* AF2653 strain. The test and control cultures were further supplemented with final concentrations of 1.95-250 µg/mL of M20 fraction or DMSO, respectively. The final DMSO concentration in all cultures was 0.5%. The culture tubes were incubated for 5 days in the dark at 25°C. Fungus disks (mycelium) were then scraped off the tubes, filtered remove all culture medium and placed into clean micro-centrifuge tubes for weighing. Aflatoxins were extracted from 500 µL of filtered culture medium using chloroform, derivatized with trifluoroacetic acid and quantified with HPLC equipped with absorption and fluorescence detectors as described previously [20]. The experiments were performed as independent triplicates (i.e. three biological replicates) at each M20 concentration. IC_{50} values for AFB1 production were obtained using equations (3) and (4) as described in the main text.

1.4. RNA isolation for RNA-seq analysis

Fungal mycelia were harvested with sterile spatulas to fresh micro-centrifuge tubes and grounded to fine powder in liquid nitrogen. The cell mass was supplemented with 1 µl (40 U/µl) of X2500 RiboLock RNase inhibitor (ThermoFisher Scientific) and immediately used to extract RNA with Trizol Reagent according to the manufacturer´s instructions. The pure total RNA was dissolved in TE buffer [10 mM Tris-HCl (pH 8.0), 1 mM EDTA] [30]. Multiple quality controls were applied to the isolated RNA. RNA degradation and contamination were monitored on 1% agarose gels. RNA purity, i.e., A260/A280 and A260A/230 ratios, was checked using the NanoPhotometer spectrophotometer (Implen, CA,

USA). Finally, RNA integrity analysis and quantitation were done using the RNA Nano 6000 Assay Kit of the Bioanalyzer 2100 system (Agilent Technologies, CA, USA). RNA samples used for RNA-seq had RNA integrity numbers (RIN) 8.6–9.4.

Table S1. Primers used to amplify ITS2 and ETS regions in *Z. bungeanum* genome

**rDNA region**	**Sequence**
ITS2-F	ATGCGATACTTGCTGTGAAT
ITS2-R	GACGCTTCTCCAGACTACAAT
ETS-F	ATAGAGCGCGTGAGTGGTG
ETS-R	GACAAGCATATGACTACTGGCAGGATCAA

Table S2. Solvent gradients used to fractionate crude methanolic extract on silica column

Fraction	Solvent	Ratio (v/v)
F1	Hexane	1.00
F2	Hexane/di chloromethane	0.80/0.20
F3	Hexane/di chloromethane	0.50/0.50
F4	Hexane/di chloromethane	0.20/0.80
F5	di chloromethane/Ethyl acetate	0.80/0.20
F6	di chloromethane/Ethyl acetate	0.50/0.50
F7	**di chloromethane/Ethyl acetate (EA80)**	**0.20/0.80**
F8	Ethyl acetate	1.00
F9	**Ethyl acetate/Methanol (M20)**	**0.80/0.20**
F10	Ethyl acetate/Methanol	0.50/0.50
F11	Ethyl acetate/Methanol	0.20/0.80
F12	Methanol	1.00

Table S3. RT-PCR primers and their target genes

**Gene name**	**Primer sequence**	**Product length (bp)**
β-tub	F: AACGTCTACTTCAACGAGGCCA<br>R: GTACCAGGCTCAAGATCAACGAG	74
hypC	F: GGTCCTTTTTGACGGGAGCC<br>R: TCTCGATAAGAATGGGAATGGTGA	63
aflW	F: CGATGTCTTTGTGCGGACG<br>R: GTGTTGCCCGCTAGCACTC	51
aflN	F: CAAGGCGAGGTGTTTCCTCT<br>R: GGCAAGTGGGTGATCCTTGA	114
aflQ	F: GGGAGGATCGGACACGACA<br>R: CATGGCCACAAAAAAGCTAGAC	52
aflR	F: CCTTTCTCACTACTCGGGTTT<br>R: GCAGGTAATCAATAATGTCGG	88
aflS	F: CTCGATGCGGCAGTGTATCT<br>R: ACACCTCCACATGAGCCTTG	109
veA	F: CGTCAGCCGGATCACTCG<br>R: GACGGTCCGCAGAGGACTT	113
ppoC	F: GTTCACAATCAGGCTCAAATGTTC<br>R: CAGGCAGTAGCGCATCAACTT	138
cat2	F: TGGCAGCAGTGACGGAAAG<br>R: AGCCCAAGCGGCAACAA	55
brlA	F: TCTAGCGGGGATGACCTCAA<br>R: CCGAAGGAAGCCAAAAGTGC	131

Table S4. High-resolution mass spectrometry-based identification of compounds in M20 and EA80 fractions

Compound	Detected fraction	Molecular formula	Calculated mass (m/z) (Theoretical)	Measured mass (m/z) (Observed)	Mass error (Δ) (ppm)
Quercetin	M20 & EA80	$C_{15}H_{10}O_7$	301.03483 (M-H)	301.03384 (M-H)	3.32
Epicatechin	M20 & EA80	$C_{15}H_{14}O_6$	289.0790 (M-H)	289.00769 (M-H)	0.63
Kaempferol-3-O-rhamnoside	M20 & EA80	$C_{21}H_{20}O_{10}$	431.097282 (M-H)	431.09574 (M-H)	4.83
Hyperoside	M20	$C_{21}H_{20}O_{12}$	463.08765 (M-H)	463.08474 (M-H)	6.27

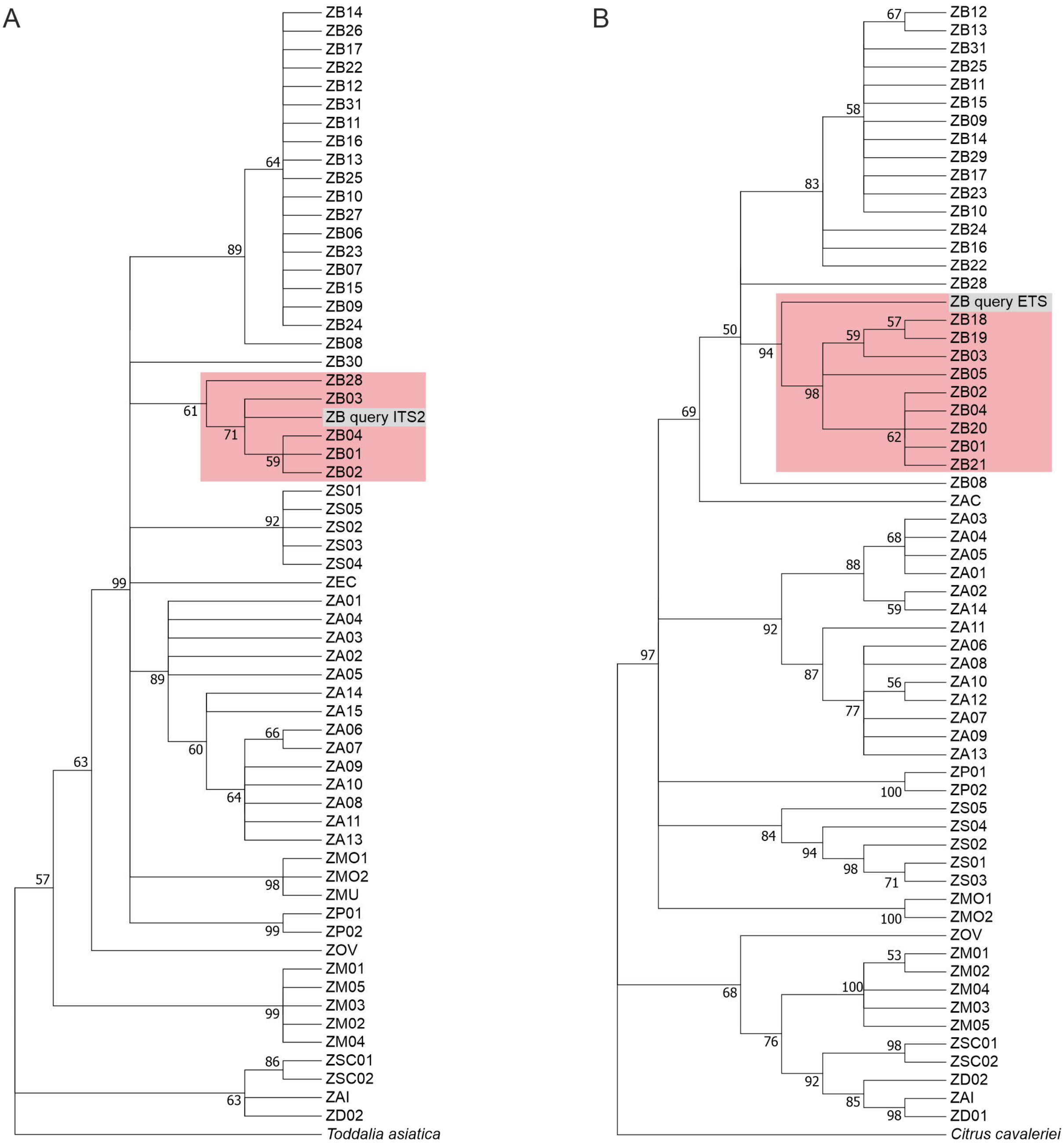

C

Query sequence	Alignment score (bits)	Expect value	Identities	Gaps	Top Blast hit	GenBank accession no.
ITS2	414	2e-111	224/224 (100%)	0/224	*Z. bungeanum* isolate KPS0225A04	MF096856.1
	414	2e-111	224/224 (100%)	0/224	*Z. bungeanum* voucher YC0167MT63	KP642589.1
ETS	815	0	447/450 (99.3%)	0/450	*Z. bungeanum* voucher B41	MF070123.1

Figure S1. Confirmation of the identity of *Zanthoxylum bungeanum* plant. Consensus neighbour-joining tree of (A) ITS2 and (B) ETS nucleotide sequences was constructed using MEGA 7.0 [22]. Algorithm parameters: 500 replicates, Kimura 2-parameter substitution model, gamma site rate variation (shape parameter=1). The percentage of replicate trees in which the associated taxa clustered together in the bootstrap test are shown adjacentt to the branches; if this value was <50%, the branches were collapsed. All ambiguous positions were removed for each sequence pair. There were 229 (ITS2) or 454 (ETS) sequence positions in the final datasets, respectively. The ITS2 and ETS trees were rooted to *Toddalia asiatica* and *Citrus cavaleriei* outgroups, respectively. Reference ITS2 and ETS sequences are from validated *Zanthoxylum* species (identified by letters) and cultivars (numbers) as described in [21]. Species abbreviations used: ZB, *Z. bungeanum*; ZA, *Z. armatum*; ZP, *Z. piperitum*; ZS, *Z. simulans*; ZM, *Z. micranthum*; ZSC, *Z. scandens*; ZMO, *Z. molle*; ZAI, *Z. ailanthoides*; ZAC, *Z. acanthopodium*; ZEC, *Z. echinocarpum*; ZMU, *Z. multijugum*; ZD, Z. dissitum; ZOV, *Z. ovalifolium*. Query sequences (gray rectangles), which were obtained from the plant material used in this study, formed monophyletic groups (pink rectangles) with validated *Z. bungeanum* cultivars in both ITS2 and ETS trees. (C) The standard nucleotide BLAST [23] search using our ITS2 and ETS sequences found validated *Z. bungeanum* sequences the best hits from GenBank nucleotide collection [24].

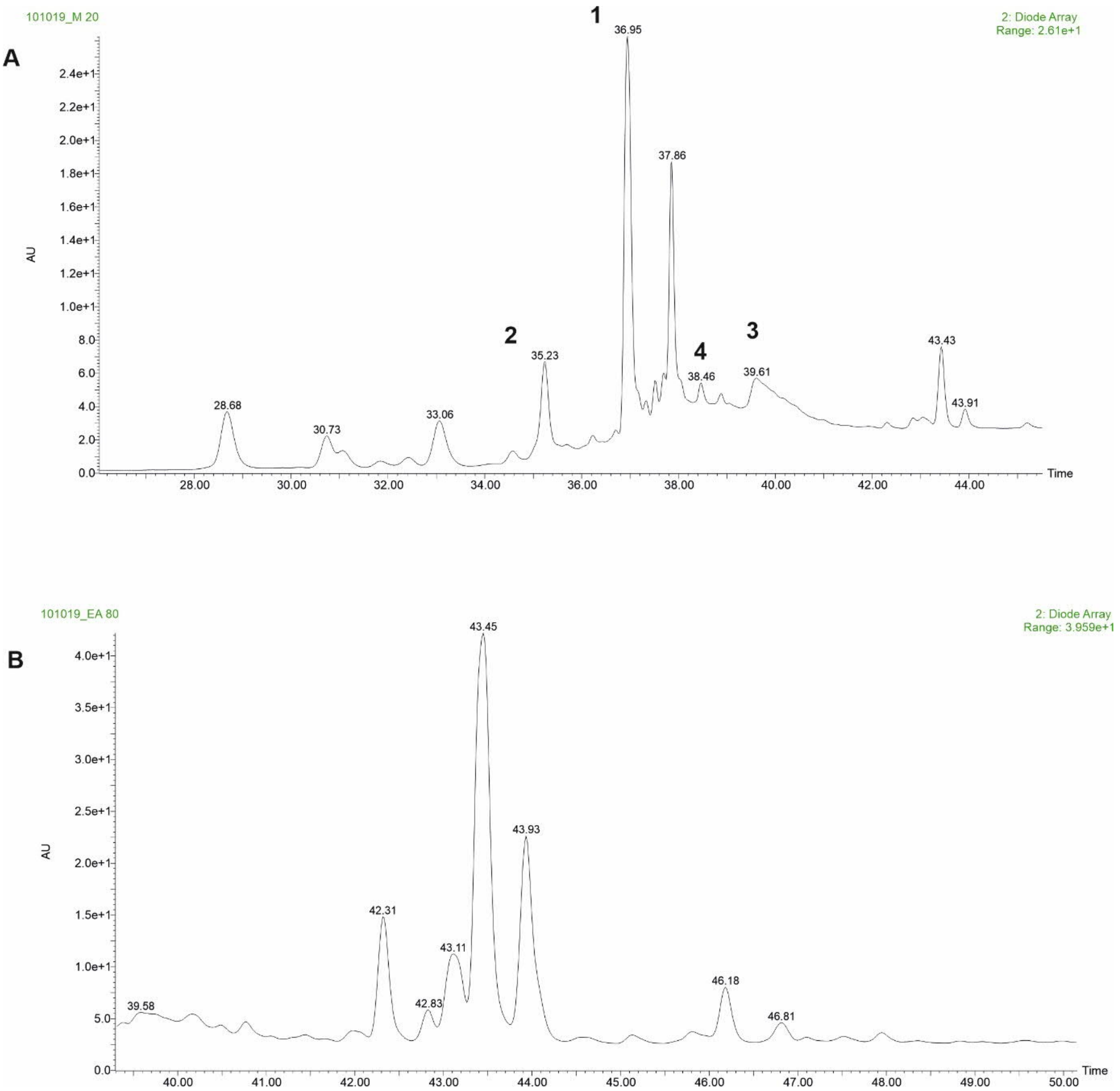

Figure S2. HPLC analysis of plant extract fractions. Chromatograms show the separation of major compounds in fractions (A) M20 and (B) EA80. Peak identifications: 1, quercetin; 2, epicatechin; 3, kaempferol-3-O-rhamnoside; 4, Hyperoside. Retention times in minutes are indicated on top of each peak.

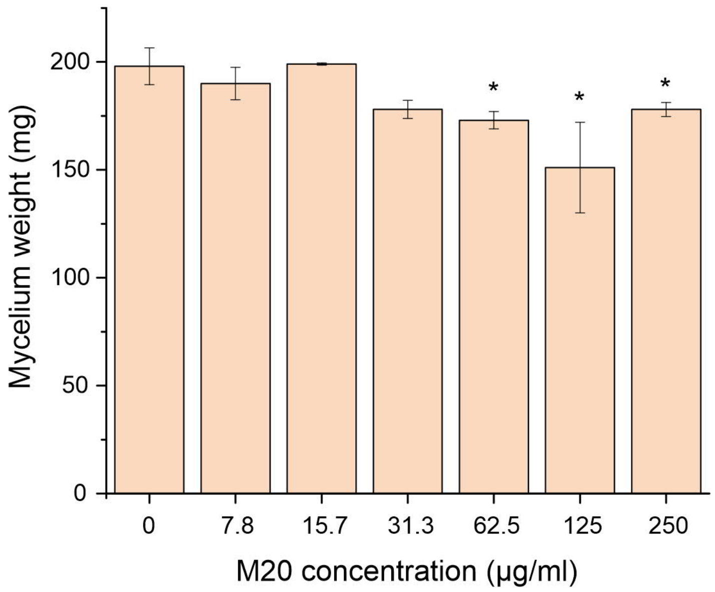

Figure S3. Effect of M20 fraction on mycelium growth. Mycelium weights from *A. flavus* cultures grown in the presence of different concentrations of M20 fraction are shown. Data are presented as mean ± S.D (n=3). ANOVA rejected the null hypothesis that all extract concentrations behaved the same ($p < 0.001$). The samples showing significant difference ($p < 0.05$) to 0 µg/ml M20 control in Tukey's HSD test are indicated with asterisk.

A

Sample name	Raw reads	Clean reads	Raw bases	Clean bases	Error rate(%)	Q20(%)	Q30(%)	GC content(%)
control 1	26999858	26556925	8.1G	8.0G	0.03	97.93	94.33	51.93
control 2	32452414	32120984	9.7G	9.6G	0.02	98.27	95.15	52.04
control 3	25253457	24881283	7.6G	7.5G	0.02	98.33	95.26	52.02
test 1	24802737	24487498	7.4G	7.3G	0.02	98.06	94.69	51.95
test 2	27658590	27298611	8.3G	8.2G	0.02	98.18	94.97	52.00
test 3	31126443	30717672	9.3G	9.2G	0.02	98.08	94.68	52.10

B

Sample name	Control 1	Control 2	Control 3	Test 1	Test 2	Test 3
Total reads	53113850	64241968	49762566	48974996	54597222	61435344
Total mapped reads	48422762	57132167	42092843	44133244	49801381	56016083
Uniquely mapped reads	48192863	56417058	41187513	43694898	49495536	55659146
Multiple mapped reads	229899	715109	905330	438346	305845	356937
Total mapping rate	91.17%	88.93%	84.59%	90.11%	91.22%	91.18%
Uniquely mapping rate	90.74%	87.82%	82.77%	89.22%	90.66%	90.60%
Multiple mapping rate	0.43%	1.11%	1.82%	0.90%	0.56%	0.58%

C

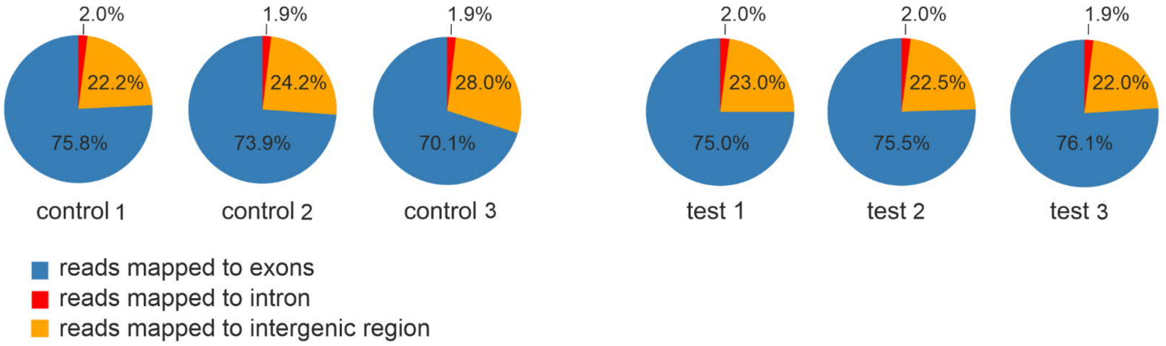

D

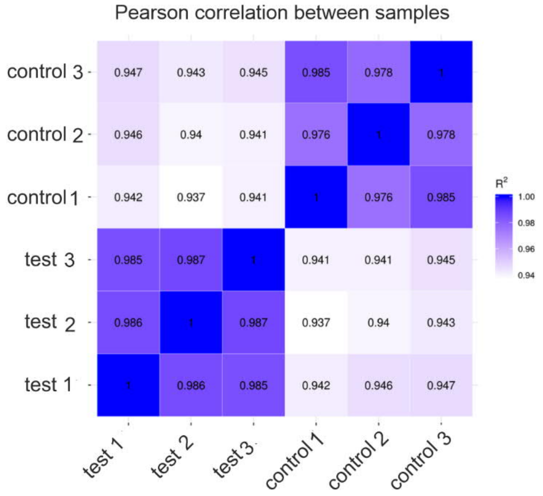

Figure S4. Quality control statistics of RNA-seq data. (A) Read counts, read quality, and observed GC content of individual control and treated samples. (B) Reads mapping statistics using *A. flavus* NRRL 3357 as the reference genome. (C) Mapping of reads to exons, introns, and intergenic regions, respectively. (D) Pearson correlation between all test and control samples. R^2 is square of Pearson correlation coefficient.

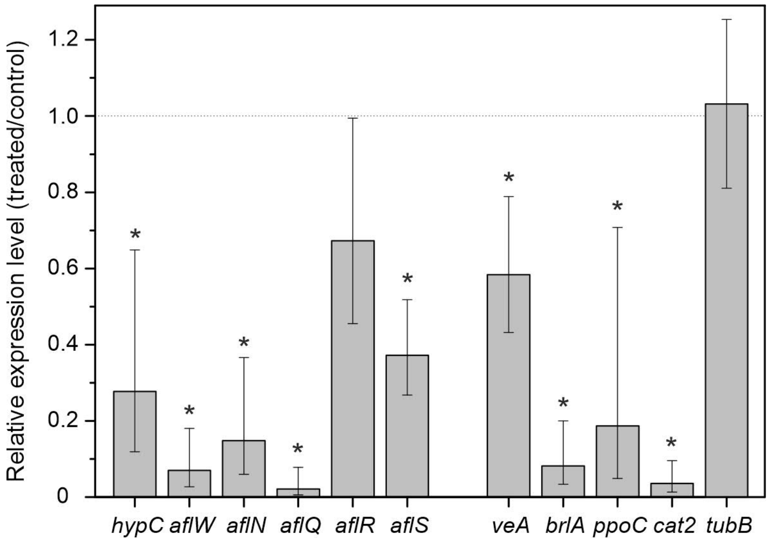

Figure S5. RT-PCR based verification of gene expression changes. Statistically significant changes are indicated with asterisk (a two-sided *t*-test, $p < 0.05$).
